# A novel adenovirus vector for easy cloning in the E3 region downstream of the CMV promoter

**DOI:** 10.1186/1743-422X-5-73

**Published:** 2008-06-06

**Authors:** Laurent Mailly, Charlotte Boulade-Ladame, Georges Orfanoudakis, François Deryckere

**Affiliations:** 1 Unité Mixte de Recherche 7175, Ecole Supérieure de Biotechnologie de Strasbourg, Illkirch, France; 2 Unité Inserm 748, Institut de Virologie, Strasbourg, France

## Abstract

The construction of expression vectors derived from the human adenovirus type 5 (Ad5), usually based on homologous recombination, is time consuming as a shuttle plasmid has to be selected before recombination with the viral genome. Here, we describe a method allowing direct cloning of a transgene in the E3 region of the Ad5 genome already containing the immediate early CMV promoter upstream of three unique restriction sites. This allowed the construction of recombinant adenoviral genomes in just one step, reducing considerably the time of selection and, of course, production of the corresponding vectors. Using this vector, we produced recombinant adenoviruses, each giving high-level expression of the transgene in the transduced cells.

## Findings

The most commonly used method for generating recombinant adenoviral vectors is based on homologous recombination in *E. coli *[1,2]. This method requires a first cloning step into a shuttle plasmid containing a promoter for the expression of the transgene. After selection of recombinants, the plasmid DNA of each clone has to be transferred into an other bacterial strain (i.e. DH5α, DH10b) because the homologous recombination is performed with BJ5183 *E. coli *strain [3] that does not allow for production of large quantities of plasmid. Improvements to this method have been made by using Top10F' bacteria that produce a high copy number of plasmids [4] or *in vitro *ligation for the subcloning of a gene of interest in the viral genome [5-7]. However, although these techniques allow efficient generation of recombinant adenoviral genomes, two-step plasmid manipulation is necessary.

**Figure 1:**
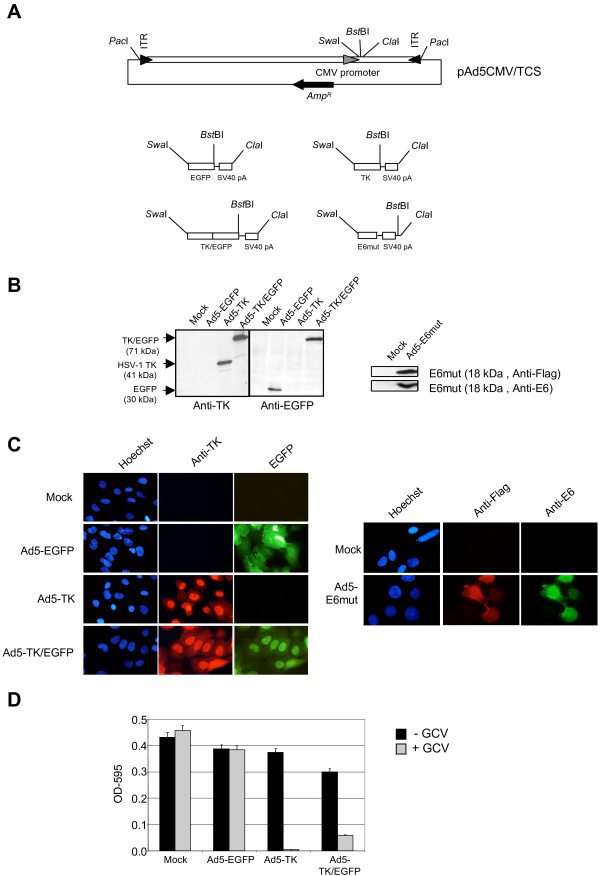
**Expression of EGFP, TK, TK/EGFP and E6mut in HeLa cells after transduction with Ad5-EGFP, Ad5-TK, Ad5-TK/EGFP and Ad5-E6mut. **(A) Restriction map of pAd5CMV/TCS and of the 4 different inserts. (B) HeLa cells were seeded on 24 well plates (1.2 × 10^5 ^cells/well) and transduced the next day with the different Ad vectors at a MOI of 1000. The TK/EGFP encoded fusion protein consisted of the entire TK protein at the N-terminus, a peptide linker SFKST and the complete EGFP protein at the C-terminus. Western-blotting analyses were carried out as described, using rabbit anti-TK antibody (obtained from William C. Summers, Yale University, New Haven, dilution 1/1300), mouse anti-EGFP antibody (Roche Diagnostics, dilution 1/1000), rabbit anti-Flag antibody (Sigma, dilution 1/2000) or mouse anti-HPV16 E6 protein antibody (1/500) [11]. (C) HeLa cells were seeded on 24 well plates and transduced as described above. Cells were fixed and treated for immunofluorescence microscopy as described [11] with anti-TK antibody (1/1300), with anti-Flag antibody (1/1000), or with anti-E6 antibody (1/1000) and with a goat anti-rabbit antibody coupled to Alexa 568 (Molecular Probes, dilution 1/1000) or goat anti-mouse antibody coupled to Alexa 488 (Molecular Probes, dilution 1/1000). The nuclei were stained with Hoechst 33342 for 5 min at room temperature. Cells were viewed using a Zeiss Axioplan microscope (D) Cells were seeded and transduced as described above. Forty-eight hours after infection, cells were incubated, or not, with ganciclovir (GCV) at 20 μg/mL. Four days later, surviving cells were analyzed using the MTT test (M2003, Aldrich-Sigma, St Quentin Fallavier, France) as described previously [12]. This test was performed in triplicates, error bars are standard deviations.

Here, a simple approach for generating an Ad5 derived expression vector is described. The first step was to construct pAd5CMV/TCS, a plasmid containing the Ad5 genome deleted of E1 and containing the immediate early CMV promoter (CMVp) upstream of a triple cloning site (TCS) composed of three unique restriction sites (*Swa*I, *Bst*BI, *Cla*I) in replacement of the E3 region. To obtain pAd5CMV/TCS, two DNA fragments surrounding the E3 region were PCR-amplified (for primers see Table 1), using pTG3622 [1] as template, and sequentially cloned on either side of the CMVp in pcDNA3 to give the pLeft/Right plasmid. Thereafter, annealed oligonucleotides, containing the TCS, were inserted into the *Bam*HI/*Not*I opened pLeft/Right to obtain the pLeft/Right/TCS. This plasmid was then used to replace the E3 region by the CMVp and TCS, in pTG3622, using homologous recombination in *E. coli *as previously described [1] to obtain pAd5CMV/TCS (Fig. 1A).

**Table 1:** Oligonucleotides used in this study (restriction sites are in bold)

Oligonucleotides used for:	5'-3' sequences	length of amplified fragments (bp)
Amplification of E3 flanking regions	CGCG**ACGCGT**TTCGACAGGGCTAC	
	CGCG**ACGCGT**GTTTCAGGCGCAGTTG	2731
	CCCTAGA**TCTAGA**AATGGACG	
	GCG**TCTAGA**TCCAATATTCTGGGTCC	2013
Insertion of TCS in adenovirus genome	GATAACAG**ATTTAAAT**CC**TTCGAA**CAGA**ATCGAT**	
	GGCC**ATCGAT**TCTG**TTCGAA**GG**ATTTAAAT**CTGTT	
PCR to check pAd5CMV/TCS	CGTGTCATATGGATACACGGG	
	TCCAGCATGGCTACAACCTC	2643
EGFP amplification from pEGFPC3	AGGAAAAAA**ATTTAAAT**CCACCATGGTGAGCAAGGGCGAGGAGCT	
	AGGAAAAAA**ATCGAT**CGCGTTAAGATACATTGAGTTTGGAC	1034
PCR to check pAd5CMV-EGFP	GGCACCAAAATCAACGGGAC	
	AGGAAAAAAATCGATCGCGTTAAGATTACATTGAGTTTGGAC	2312
Amplification of TK from pMBP-TK	AGGAAAAAA**ATTTAAAT**GCGCGTATGGCTTCGTAC	
	AGGAAAAAA**ATTTAAAT**GAGTTAGCCTCCCCCATC	1129
	AGGAAAAAA**TTCGAA**TCAGTTAGCCTCCCCCATC	1129

Four different constructs were inserted into pAd5CMV/TCS to drive the expression of either the enhanced green fluorescent protein (EGFP), thymidine kinase from Herpes Simplex Virus type 1 (TK), a TK/EGFP fusion protein or a mutated form of the HPV16 E6 protein (Fig. 1A) [8]. The EGFP ORF and the SV40 polyadenylation site from the pEGFP-C3 (Clontech, Saint-Germain en Laye, France) was inserted using the *Swa *I and *Cla *I restriction sites after PCR amplification (primers are listed in Table 1). For the resulting plasmid, pAd5-EGFP, it is possible to exchange the EGFP ORF (using *Swa*I and *Bst*BI) while keeping the SV40 polyadenylation site (Fig 1A).

The TK ORF was PCR-amplified from pMBP-TK [9] and inserted into pAd5-EGFP either in replacement of the EGFP ORF (*Swa*I-*Bst*BI) or fused upstream of the EGFP coding sequence (*Swa*I). E6mut, a flag-tagged dominant negative mutant of HPV16 E6 protein (E6-6C/6S-F47R-ΔPDZ), was also successfully sub-cloned [8]. This was achieved by inserting a *Klenow*-repaired *Eco*RI fragment containing the E6mut ORF into the *Swa*I site of pAd5CMV/TCS. For each construction, a good ratio of positive clones was obtained, respectively 14/20, 6/20, 12/20 and 3/10, in only three days (from the start of cloning until the plasmid preparation and restriction verification).

The four corresponding recombinant adenoviruses were produced in 293 cells following classical procedures [10] and tested on HeLa cells. The expression of the different proteins was examined by Western blotting (Fig 1B), EGFP fluorescence and immunofluorescence (Fig 1C). This demonstrated that (i) cells are were efficiently transduced, (ii) the fusion protein TK/EGFP conserved the green fluorescence conferred by its EGFP moiety, (iii) the fusion TK/EGFP conserved the TK epitopes, and (iv) the E6 mutant protein was well expressed and recognized by both anti-E6 and anti-Flag antibodies. Cells expressing TK are sensitive to the pro-drug ganciclovir and the ability of the fusion product to induce cell death was investigated using a Methylthiazolyldiphenyl-tetrazolium bromide (MTT) assay (Fig. 1D). This test demonstrated that both TK and TK/EGFP induced the death of HeLa cells after treatment with ganciclovir.

This approach can be easily used in any laboratory to rapidly produce recombinant adenoviruses. For this, a new vector derived from Ad5 has been created by inserting, in replacement of the E3 region, the CMVp followed by three unique restriction sites (*Swa*I, *Bst*BI,*Cla*I) that are absent from a ΔE1 Ad5 genome. This triple cloning site allows the easy cloning of a transgene that will be expressed from the CMVp. In addition, pAd5-EGFP, allows the cloning a cDNA of interest between the CMV promoter and the SV40 polyadenylation signal either in replacement of the EGFP ORF or in fusion with it. This is also possible with this vector to clone a second transgene in the E1 region by using homologous recombination in *E. coli *as previously described [2]. Four different transgenes were inserted into pAd5CMV/TCS. The construction of the corresponding genomes, contained in plasmids, was rapidly achieved (3 days instead of 7 to 10 days with homologous recombination in *E. coli*). In each case, a much higher proportion of positive clones after ligation into pAdCMV5-TCS (30–70%) was obtained when compared to homologous recombination (15% at best in our laboratory). The constructs led to the production of infectious adenoviral particles that allowed the high-level expression of the different transgenes. This method of construction of adenovirus vectors is clearly faster and easier than conventional approaches and can be used by those who are not familiar with the homologous recombination system.

## Competing interests

The authors declare that they have no competing interests.

## Authors' contributions

Design and conception of study (FD, GO); plasmid constructs and production of viruses (LM); gene expression analysis (LM, CB-L); manuscript preparation (LM, FD). All authors read and approved the final manuscript.
